# The clinical value of inflammation index in predicting ICU mortality of critically ill patients with intracerebral hemorrhage

**DOI:** 10.3389/fpubh.2024.1373585

**Published:** 2024-08-02

**Authors:** Guang Zhao, Yuting Gu, Zhaoxiang Wang, Yuyang Chen, Xiaohua Xia

**Affiliations:** ^1^Department of Emergency Medicine, The First People’s Hospital of Kunshan, Kunshan, China; ^2^Department of Endocrinology, The First People’s Hospital of Kunshan, Kunshan, China

**Keywords:** ICH, NLR, PLR, LMR, SII, SIRI, ICU mortality

## Abstract

**Background:**

The inflammatory response holds paramount significance in the context of intracerebral hemorrhage (ICH) and exhibits a robust correlation with mortality rates. Biological markers such as the neutrophil-to-lymphocyte ratio (NLR), platelet-to-lymphocyte ratio (PLR), lymphocyte-to-monocyte ratio (LMR), systemic immune inflammation index (SII), and systemic inflammatory response index (SIRI) play crucial roles in influencing the systemic inflammatory response following ICH. This study aims to compare the predictive efficacy of NLR, PLR, LMR, SII, and SIRI concerning the risk of mortality in the intensive care unit (ICU) among critically ill patients with ICH. Such a comparison seeks to elucidate their early warning capabilities in the management and treatment of ICH.

**Methods:**

Patients with severe ICH requiring admission to the ICU were screened from the Medical Information Marketplace for Intensive Care (MIMIC-IV) database. The outcomes studied included ICU mortality and 30 day ICU hospitalization rates, based on tertiles of the NLR index level. To explore the relationship between the NLR index and clinical outcomes in critically ill patients with ICH, we utilized receiver operating characteristic (ROC) analysis, decision curve analysis (DCA), and multivariate logistic regression analysis.

**Results:**

A total of 869 patients (51.9% male) were included in the study, with an ICU mortality rate of 22.9% and a 30 day ICU hospitalization rate of 98.4%. Among the five indicators examined, both the ROC curve and DCA indicated that NLR (AUC: 0.660, 95%CI: 0.617–0.703) had the highest predictive ability for ICU mortality. Moreover, this association remained significant even after adjusting for other confounding factors during multivariate analysis (HR: 3.520, 95%CI: 2.039–6.077). Based on the results of the multivariate analysis, incorporating age, albumin, lactic acid, NLR, and GCS score as variables, we developed a nomogram to predict ICU mortality in critically ill patients with ICH.

**Conclusion:**

NLR emerges as the most effective predictor of ICU mortality risk among critically ill patients grappling with ICH when compared to the other four indicators. Furthermore, the integration of albumin and lactic acid indicators into the NLR nomogram enhances the ability to promptly identify ICU mortality in individuals facing severe ICH.

## Introduction

Spontaneous intracerebral hemorrhage (ICH) represents a prevalent subtype of stroke characterized by elevated morbidity, substantial disability, and heightened mortality rates. This condition poses a significant threat to the lives and well-being of over 3 million individuals globally each year (1). With the incidence of ICH increasing annually, it imposes a considerable burden on patients, their families, and society as a whole (2, 3). Even with interventions such as early hematoma removal surgery, blood pressure control, and hemostasis, the mortality rate associated with ICH remains alarmingly high, reaching up to 68% (4).

Indeed, ICH is recognized not only as a localized brain condition but also as a systemic disease impacting multiple organ systems. A growing body of evidence underscores the pivotal role played by systemic inflammation and immune responses in the pathophysiological processes of ICH (5). Brain damage caused by ICH triggers an inflammatory immune response. This response, mediated through the sympathetic nervous system and the hypothalamus-pituitary-adrenal (HPA) axis, leads to persistent inflammation, reducing systemic immune activity and inhibiting cellular immune response. Consequently, this exacerbates the condition of patients with ICH and significantly impacts their prognosis. White blood cell counts and its subtypes counts are associated with hematoma enlargement, mortality, and unfavorable prognosis in ICH (6, 7). The Neutrophil-to-Lymphocyte Ratio (NLR) has shown promise as a valuable biomarker for predicting the prognosis of stroke (8). Furthermore, the NLR and Platelet-to-Lymphocyte Ratio (PLR) have been identified as crucial indicators of prognosis in patients with ICH (9). The lymphocyte to monocyte ratio (LMR) may be associated with the long-term neurological prognosis of acute ischemic stroke and can serve as a supplementary marker for the diagnosis of chronic heart failure (10, 11). The systemic inflammatory response index (SIRI), derived from neutrophil, monocyte, and lymphocyte counts, stands as a novel systemic inflammatory marker. It has been identified as an independent prognostic indicator for various tumors and could predict the risk of aneurysmal subarachnoid hemorrhage (SAH) (12–14). The systemic immune-inflammation index (SII) is a novel biomarker for malignancy and inflammatory diseases, calculated based on platelet, neutrophil, and lymphocyte counts. It has been used to predict adverse outcomes following cerebral hemorrhage and cerebral vasospasm in late aneurysmal SAH patients (15, 16). Additionally, SII has proven useful in identifying high-risk groups for poor prognosis in chronic kidney disease and abdominal aortic calcification, thereby aiding in preventive measures for the general population (17, 18). Despite the existence of immune inflammation-related biomarkers, their predictive capabilities regarding the mortality of patients with ICH remain relatively insufficient. This limitation hinders their ability to serve as effective warning indicators in treatment decisions.

Therefore, the primary objective of this study was to assess the predictive capability of NLR, PLR, LMR, SIRI, and SII values upon admission for ICU mortality in patients with ICH. Additionally, the study aimed to explore the potential application of these markers in the early identification of ICH severity.

## Methods

### Study population

**Figure 1:**
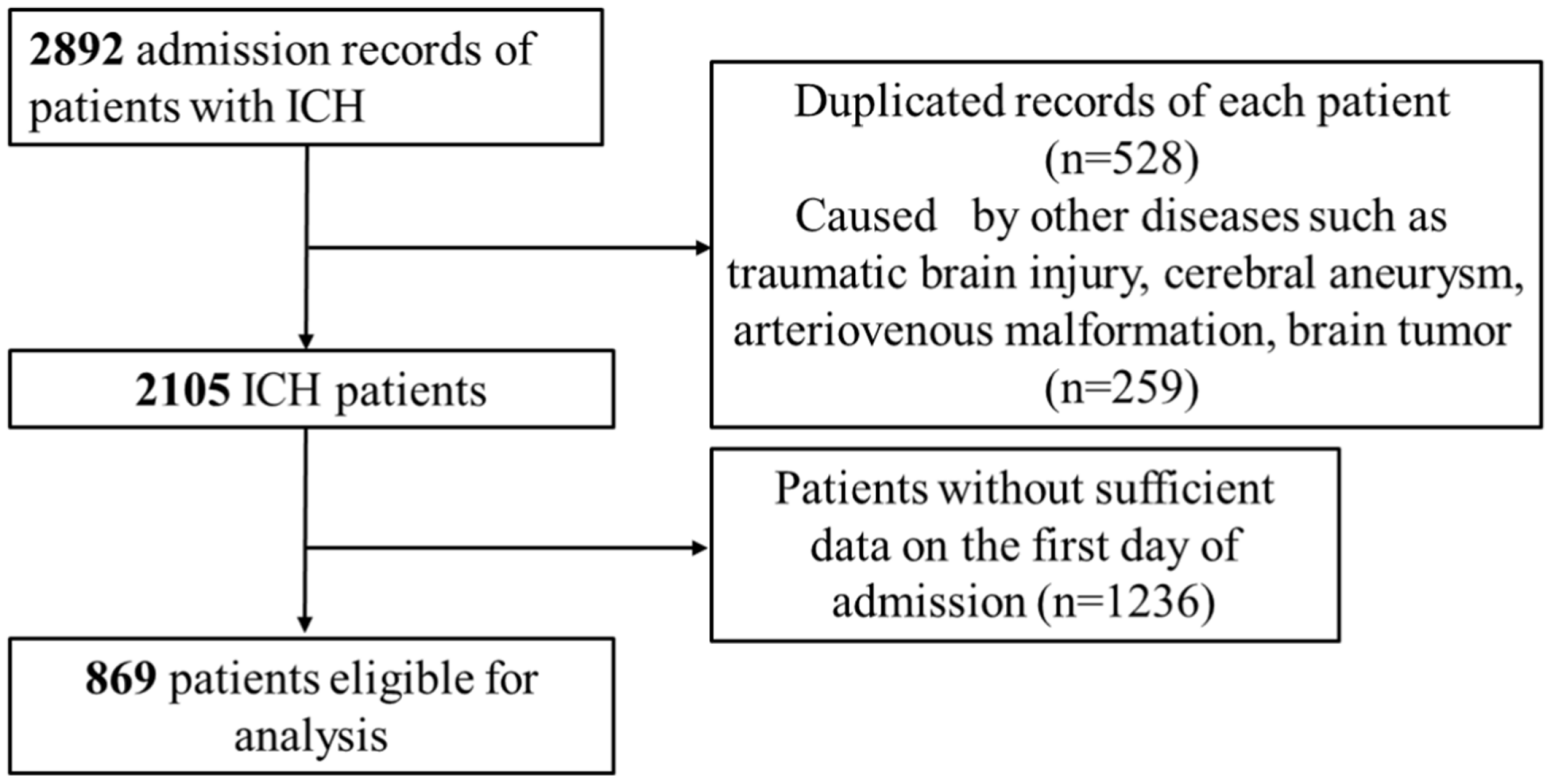
The flowchart of the study.

The current investigation utilized health-related data from the MIMIC-IV (version 2.2) repository, a widely-used extensive database developed and overseen by the MIT Computational Physiology Laboratory (19). GZ, a researcher, adhered to all access requirements and conducted the data extraction. This study included patients diagnosed with ICH based on the International Classification of Diseases, 9th and 10th Revision (ICD code: ICD-9th: 431, 7,670, 7,721; ICD-10th: I61, I62). Specific exclusion criteria were applied as follows: (1) individuals under the age of 18 upon their initial admission; (2) patients with ICH caused by traumatic brain injury, brain tumor, cerebral aneurysm, or cerebral arteriovenous malformation; (3) patients with multiple admissions for ICH, with data extracted only from the first admission; (4) patients with severe liver and kidney conditions, leukemia, lymphoma, other hematological diseases, or malignant tumors (to eliminate the potential bias caused by abnormal hematology indices on the study results); (5) patients lacking sufficient data, specifically Neutrophil and Lymphocyte counts, on the first day of admission. In total, 869 patients were enrolled in this research and categorized into three groups according to the tertiles of the NLR index (Figure 1).

### Data collection

The information extraction process utilized PostgreSQL (version 14.10) software and Navicat Premium (version 16), with Structured Query Language (SQL) being the tool for extraction. The potential variables were grouped into four main categories: (1) demographic variables, including age, race, sex, and weight, (2) comorbidities, encompassing hypertension, diabetes, and hyperlipemia, (3) laboratory indicators, consisting of red blood cells (RBC), white blood cells (WBC), hemoglobin (Hb), platelet (PLT), neutrophil, lymphocyte, monocyte, lactic acid, and albumin, (4) the Glasgow Coma Scale score (GCS), with lower scores indicating a worse level of consciousness. Patients with ICH were stratified into three groups based on GCS scores: 13–15 for mild coma, 9–12 for moderate coma, and 3–8 for severe coma. Equations were applied to calculate various parameters, such as NLR, PLR, SII, and SIRI, as follows: NLR = count of neutrophils/count of lymphocytes; PLR = count of platelets/count of lymphocytes; LMR = count of lymphocytes/count of monocytes; SIRI = (count of neutrophils × count of monocytes)/count of lymphocytes; and SII = (count of neutrophils × count of platelets)/count of lymphocytes. All laboratory variables and disease severity scores were obtained from data generated within the initial 24 h of patient admission.

### Statistical analysis

Continuous variables were presented as means ± standard deviations (SDs) or medians (interquartile ranges, IQRs) and compared using Student’s *t*-test or Kruskal–Wallis rank sum test, as appropriate. Categorical variables were expressed as frequencies and percentages, and Fisher exact tests were employed for comparison when appropriate. Logistic regression analyses were conducted to assess the independent association between NLR/PLR/LMR/SIRI/SII and ICU mortality. An extended logistic model approach was applied for different covariates-adjusted models, leading to the construction of three models: Model 1, adjusted for age, race, sex, marital status, and weight; Model 2, additionally adjusted for hypertension, diabetes, and hyperlipidemia. The presence of confusion was evaluated by including or excluding covariates in the logistic regression model, and regression coefficients were compared. A significance level of two-sided *p* values <0.05 was considered statistically significant. The predictive ability of NLR/PLR/LMR/SIRI/SII was determined by calculating the area under the receiver operating characteristic curve (AUC). Receiver operating characteristic (ROC) analyses and Decision Curve Analyses (DCAs) were utilized to evaluate predictive performance. Stratified analyses were conducted to assess potential modifications of the association between NLR and ICU mortality in ICH patients by sex, marital status, race, GCS, hypertension, diabetes, hyperlipidemia, and 30 day ICU hospitalization rate. Based on the results of multivariate logistic regression analysis, a nomogram was developed to predict ICU mortality. The appropriateness of the nomogram for clinical application was determined by the calibration curve. Statistical analyses were conducted using IBM SPSS 24.0 and Empowerstats software (version 6.0).

## Results

### Baseline characteristics

**Figure 2:**
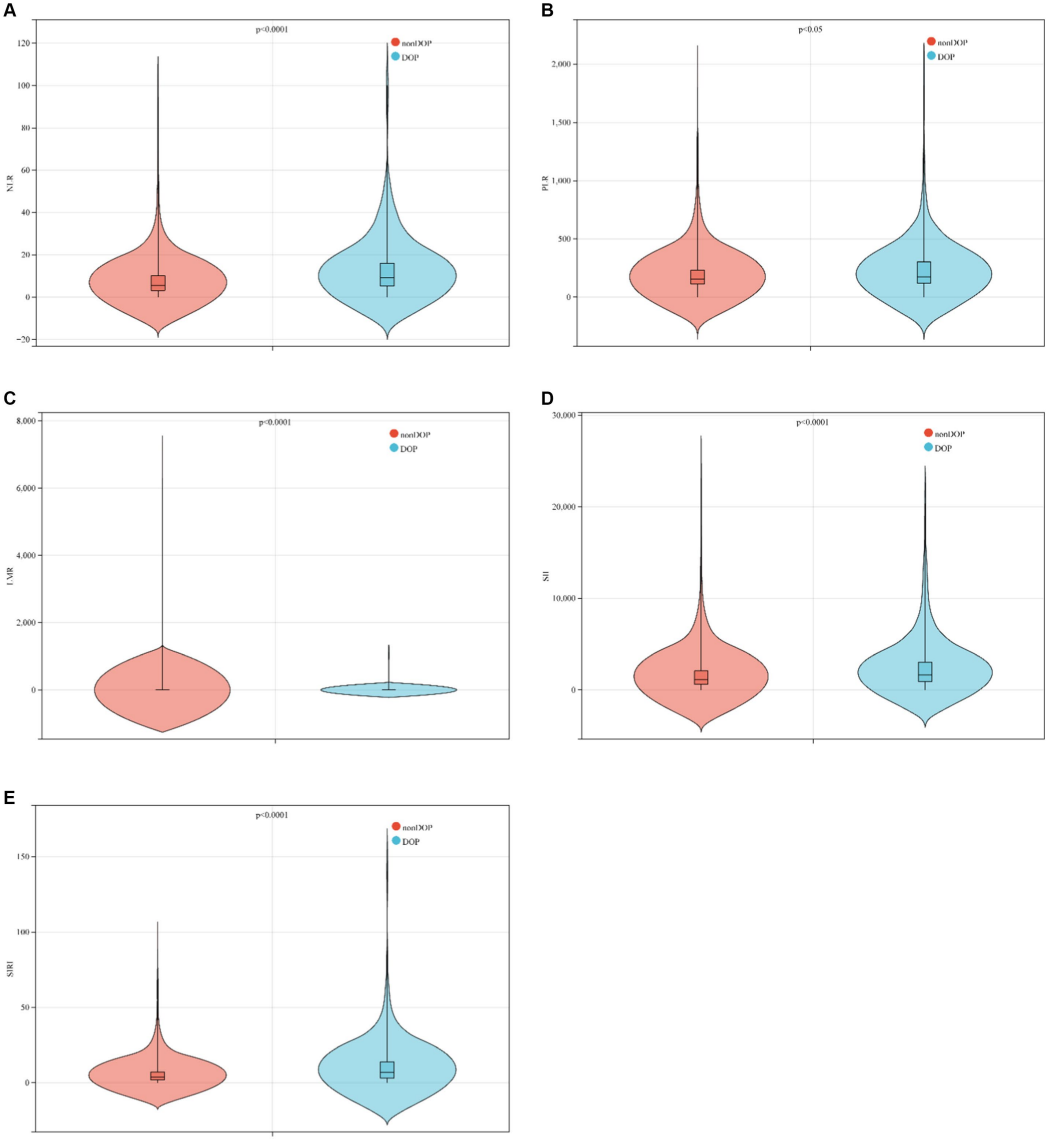
Boxplots of the NLR, PLR, LMR, SII and SIRI showing the distribution in the survivor group and non-survivor group. **(A)** The NLR of the non-survivor group was higher than that of the survivor group (*p* < 0.0001); **(B)** the PLR of the non-survivor group was higher than that of the survivor group (*p* < 0.05); **(C)** the LMR of the non-survivor group was lower than that of the survivor group (*p* < 0.0001); **(D)** the SII of the non-survivor group was higher than that of the survivor group (*p* < 0.0001); **(E)** the SII of the non-survivor group was higher than that of the survivor group (*p* < 0.0001).

This study included a total of 869 ICU patients with ICH, divided into 2 groups based on ICU death. The baseline characteristics of ICH patients are detailed in Table 1. The average age was 69.5 years (60.3–81.9 years), with 451 cases (51.9%) being male. Within the total sample, there were 199 ICU deaths (22.9%) and 852 patients requiring 30 day ICU hospitalization (98.4%). The non-survival group tended to have older individuals, higher lactic acid levels on admission, and lower GCS scores. A lower GCS score indicates poorer neurological function and a lower level of consciousness, suggesting a severe ICH and a higher risk of death. In the non-survival group, NLR (*p* < 0.001), PLR (*p* = 0.033), SII (*p* < 0.001), and SIRI (*p* < 0.001) were significantly higher compared to the survival group, while LMR (*p* < 0.001) was lower (Table 1; Figure 2).

**Table 1:** Baseline characteristics of the 869 patients with ICH.

Characteristics	Overall	Survivor	Non-survivor	*p*-value
Count	869.00	670 (77.1%)	199 (22.9%)	
Age (years)	69.506 ± 15.731	68.470 ± 16.052	72.994 ± 14.085	**<0.001**
Sex				0.964
Male	451 (51.899%)	348 (51.940%)	103 (51.759%)	
Female	418 (48.101%)	322 (48.060%)	96 (48.241%)	
Race				**<0.001**
White	501 (57.652%)	403 (60.149%)	98 (49.246%)	
Yellow	36 (4.143%)	27 (4.030%)	9 (4.523%)	
Black	100 (11.507%)	84 (12.537%)	16 (8.040%)	
Other/unknown	232 (26.697%)	156 (23.284%)	76 (38.191%)	
Marital status				0.572
Single	179 (25.176%)	149 (25.779%)	30 (22.556%)	
Married	360 (50.633%)	291 (50.346%)	69 (51.880%)	
Divorced	60 (8.439%)	51 (8.824%)	9 (6.767%)	
Widowed	112 (15.752%)	87 (15.052%)	25 (18.797%)	
Weight	74.800 (62.200–90.000)	74.300 (62.500–90.200)	75.300 (60.850–89.100)	0.604
GCS				**0.006**
Mild	744 (85.813%)	581 (86.716%)	163 (82.741%)	
Moderate	87 (10.035%)	69 (10.299%)	18 (9.137%)	
Severe	36 (4.152%)	20 (2.985%)	16 (8.122%)	
WBC (K/uL)	9.800 (7.500–13.200)	9.300 (7.300–12.600)	11.350 (8.400–15.450)	**<0.001**
RBC (m/uL)	4.220 (3.655–4.640)	4.245 (3.670–4.680)	4.130 (3.585–4.545)	**0.022**
Hb (g/dL)	12.550 (11.000–13.900)	12.600 (11.000–14.000)	12.350 (10.800–13.500)	**0.019**
PLT (K/uL)	206.000 (161.000–252.000)	211.000 (168.500–253.000)	187.500 (144.250–236.750)	**0.015**
Neutrophil (K/uL)	7.520 (5.200–10.940)	7.185 (4.962–10.107)	9.710 (6.475–13.410)	**<0.001**
Lymphocyte (K/uL)	1.220 (0.840–1.800)	1.260 (0.890–1.840)	0.970 (0.635–1.600)	**0.014**
Monocyte (K/uL)	0.690 (0.500–0.950)	0.690 (0.500–0.908)	0.730 (0.515–1.030)	**0.026**
Albumin (mg/dL)	3.800 (3.400–4.200)	3.900 (3.400–4.200)	3.750 (3.200–4.100)	**0.002**
Lactic acid (mmol/L)	1.500 (1.100–2.100)	1.400 (1.100–1.800)	1.800 (1.200–2.700)	**<0.001**
NLR	6.173 (3.485–11.326)	5.509 (3.160–10.221)	9.123 (5.384–16.063)	**<0.001**
PLR	160.145 (113.158–247.312)	154.145 (112.756–231.965)	175.532 (118.579–304.738)	**0.035**
LMR	1.799 (1.113–2.656)	1.938 (1.235–2.776)	1.367 (0.860–2.074)	**<0.001**
SII	1189.270 (666.032–2391.771)	1123.973 (623.130–2101.081)	1644.152 (904.509–3057.778)	**<0.001**
SIRI	4.280 (2.058–8.867)	3.645 (1.883–7.202)	6.862 (3.248–14.021)	**<0.001**
GCS score	13.871 ± 1.600	13.924 ± 1.366	13.690 ± 2.213	0.072
Complications				
Hypertension				0.396
No	353 (40.621%)	267 (39.851%)	86 (43.216%)	
Yes	516 (59.379%)	403 (60.149%)	113 (56.784%)	
Diabetes				0.180
No	618 (71.116%)	484 (72.239%)	134 (67.337%)	
Yes	251 (28.884%)	186 (27.761%)	65 (32.663%)	
Hyperlipidemia				0.946
No	496 (57.077%)	382 (57.015%)	114 (57.286%)	
Yes	373 (42.923%)	288 (42.985%)	85 (42.714%)	
LOS of ICU (days)				0.611
<=30	855 (98.389%)	660 (98.507%)	195 (97.990%)	
>30	14 (1.611%)	10 (1.493%)	4 (2.010%)	

ICH, intracerebral hemorrhage; WBC, white blood cell; RBC, red blood cell; Hb, hemoglobin; PLT, platelets; GCS, Glasgow coma scale; NLR, neutrophil-to-lymphocyte ratio; PLR, platelet-to-lymphocyte ratio; LMR, lymphocyte-to-monocyte ratio; SII, systemic immune-inflammation index; SIRI, systemic inflammation response index.

The *p* values < 0.05 are written in bold text.

The baseline characteristics of critically ill patients with ICH were further analyzed based on tertiles of NLR markers, as detailed in Table 2. Patients were stratified into three groups according to their NLR levels at admission: tertile (T)1 (0.46–4.14), T2 (4.15–9.22), and T3 (9.23–100). It was observed that patients in the highest tertile of NLR exhibited elevated levels of WBC, neutrophils, and monocytes, along with lower levels of lymphocytes and albumin. Furthermore, the highest NLR tertile was associated with higher ICU mortality compared to the lower group (10.345% vs. 24.221% vs. 34.138%, *p* < 0.001).

**Table 2:** Characteristics and outcomes of participants categorized by NLR.

Characteristics	Low (T1)	Middle (T2)	High (T3)	*p*-value
Count	288	291	290	
Age	68.060 ± 16.199	71.031 ± 15.284	69.411 ± 15.616	0.075
Sex				0.872
Male	153 (53.125%)	150 (51.546%)	148 (51.034%)	
Female	135 (46.875%)	141 (48.454%)	142 (48.966%)	
Race				**<0.001**
White	167 (57.986%)	172 (59.107%)	162 (55.862%)	
Yellow	10 (3.472%)	18 (6.186%)	8 (2.759%)	
Black	53 (18.403%)	34 (11.684%)	13 (4.483%)	
Other/unknown	58 (20.139%)	67 (23.024%)	107 (36.897%)	
Marital status				0.8
Single	62 (24.031%)	64 (26.446%)	53 (25.118%)	
Married	136 (52.713%)	116 (47.934%)	108 (51.185%)	
Divorced	25 (9.690%)	20 (8.264%)	15 (7.109%)	
Widowed	35 (13.566%)	42 (17.355%)	35 (16.588%)	
Weight	77.400 (66.900–94.100)	75.000 (61.000–90.000)	71.400 (60.000–85.800)	**0.002**
GCS				0.596
Mild	243 (84.375%)	250 (85.911%)	251 (87.153%)	
Moderate	34 (11.806%)	30 (10.309%)	23 (7.986%)	
Severe	11 (3.819%)	11 (3.780%)	14 (4.861%)	
WBC (K/uL)	7.900 (6.400–9.800)	9.800 (7.900–12.250)	13.200 (9.600–16.600)	**<0.001**
RBC (m/uL)	4.325 (3.822–4.685)	4.190 (3.615–4.585)	4.070 (3.580–4.657)	**0.017**
Hb (g/dL)	12.700 (11.325–14.000)	12.500 (10.900–13.950)	12.300 (10.800–13.800)	0.134
PLT (K/uL)	210.000 (165.000–246.000)	204.000 (163.500–246.000)	200.000 (155.000–269.000)	0.866
Neutrophil (K/uL)	4.750 (3.732–6.018)	7.740 (6.100–9.695)	11.945 (9.033–15.130)	**<0.001**
Lymphocyte (K/uL)	1.905 (1.412–2.388)	1.220 (0.990–1.570)	0.745 (0.510–0.998)	**<0.001**
Monocyte (K/uL)	0.640 (0.490–0.790)	0.710 (0.510–0.990)	0.745 (0.493–1.080)	**<0.001**
Albumin (mg/dL)	3.900 (3.600–4.200)	3.900 (3.400–4.200)	3.700 (3.200–4.100)	**<0.001**
Lactic acid (mmol/L)	1.500 (1.100–2.200)	1.500 (1.100–1.975)	1.500 (1.100–2.100)	0.877
PLR	106.114 (79.104–146.388)	157.364 (126.190–209.091)	280.798 (186.985–378.995)	**<0.001**
LMR	2.765 (2.115–3.867)	1.737 (1.283–2.357)	1.000 (0.706–1.492)	**<0.001**
SII	559.335 (362.238–750.141)	1247.707 (986.919–1609.776)	3036.440 (2247.421–4823.287)	**<0.001**
SIRI	1.766 (1.031–2.383)	4.466 (3.120–6.215)	11.249 (7.367–18.702)	**<0.001**
GCS score	13.969 ± 1.491	13.886 ± 1.717	13.757 ± 1.583	0.077
Complications				
Hypertension				0.745
No	115 (39.931%)	115 (39.519%)	123 (42.414%)	
Yes	173 (60.069%)	176 (60.481%)	167 (57.586%)	
Diabetes				0.078
No	193 (67.014%)	206 (70.790%)	219 (75.517%)	
Yes	95 (32.986%)	85 (29.210%)	71 (24.483%)	
Hyperlipidemia				0.28
No	162 (56.250%)	158 (54.296%)	176 (60.690%)	
Yes	126 (43.750%)	133 (45.704%)	114 (39.310%)	
LOS of ICU (days)				0.132
<=30	288 (99.310%)	285 (98.616%)	282 (97.241%)	
>30	2 (0.690%)	4 (1.384%)	8 (2.759%)	
Death of Hospital (DOH)				**<0.001**
No	260 (89.655%)	219 (75.779%)	191 (65.862%)	
Yes	30 (10.345%)	70 (24.221%)	99 (34.138%)	

NLR index: T1 (0.46–4.14); T2 (4.15–9.22); T3 (9.23–100).

### Primary outcomes

**Figure 3:**
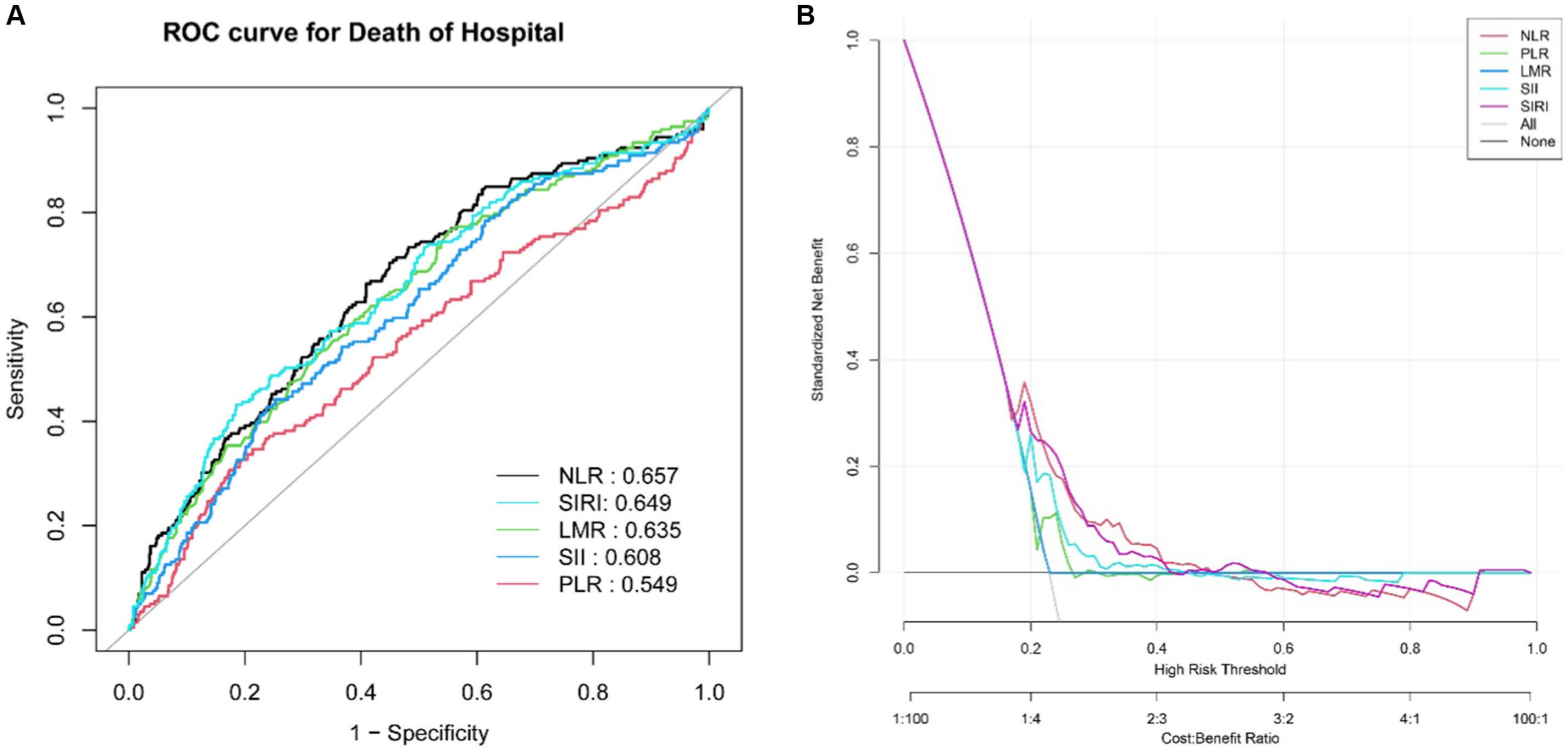
ROC curves of the NLR, PLR, LMR, SII and SIRI for predicting ICU mortality **(A)**. The DCA for evaluating the accuracy and practicability of the NLR, PLR, LMR, SII and SIRI, respectively **(B)**.

ROC analysis and DCA were employed to assess the predictive ability of the biomarkers for ICU mortality in ICH (Figure 3). AUC values were determined, revealing that NLR had the highest AUC (AUC: 0.657, 95%CI: 0.613–0.701) compared to PLR (AUC: 0.549, 95%CI: 0.501–0.597), LMR (AUC: 0.635, 95%CI: 0.591–0.680), SII (AUC: 0.609, 95%CI: 0.563–0.654), and SIRI (AUC: 0.649, 95%CI: 0.604–0.694). This suggests that NLR demonstrated a better ability to predict ICU mortality in ICH. Further details, including optimal cutoff values, specificity, and sensitivity, are provided in Table 3.

**Table 3:** AUC in predicting the risk of ICU mortality in patients with ICH.

	AUC (95%CI)	Cutoff point	Specificity	Sensitivity	PPV	NPV
NLR	0.657 (0.613–0.701)	6.665	0.5910	0.6683	0.3251	0.8553
PLR	0.549 (0.501–0.597)	262.330	0.8075	0.3266	0.3351	0.8015
LMR	0.635 (0.591–0.680)	2.105	0.4493	0.7727	0.2904	0.8649
SII	0.609 (0.563–0.654)	2038.475	0.7478	0.4422	0.3424	0.8186
SIRI	0.649 (0.604–0.694)	9.195	0.8149	0.4322	0.4095	0.8285

In the multivariate logistic regression analysis, three models were established (Table 4): an unadjusted model, a partly adjusted model (adjusted for age, sex, weight, race, and marital status), and a fully adjusted model (adjusted for age, sex, weight, race, marital status, hypertension, diabetes, hyperlipidemia, and GCS score). The results across these models consistently identified NLR, albumin, lactic acid, SII, and SIRI as noteworthy risk factors for ICU mortality in patients with ICH. When the NLR index was considered as a nominal variable, patients with a higher NLR index were associated with a significantly elevated risk of ICU mortality compared with patients in the lowest tertile of NLR index across all three established logistic proportional hazards models: Unadjusted model (HR: 4.492, 95%CI: 2.867–7.039), partly adjusted model (HR: 3.549, 95%CI: 2.076–6.068), and fully adjusted model (HR: 3.520, 95%CI: 2.039–6.077). The NLR index exhibited an increasing trend with ICU mortality (Table 4).

**Table 4:** Association between NLR and ICU mortality in multiple regression model.

Variable	Crude	*p* value	Model I	*p* value	Model II	*p* value
	HR (95% CI)		HR (95% CI)		HR (95% CI)	
NLR	1.042 (1.026, 1.058)	<0.00001	1.033 (1.015, 1.050)	0.00018	1.030 (1.013, 1.048)	0.00047
Albumin	0.660 (0.505, 0.862)	0.00233	0.568 (0.406, 0.795)	0.00095	0.624 (0.441, 0.884)	0.00795
Lactic acid	1.362 (1.136, 1.634)	0.00086	1.281 (1.044, 1.572)	0.01743	1.291 (1.053, 1.582)	0.01415
PLR	1.001 (1.000, 1.002)	0.03919	1.000 (0.999, 1.002)	0.53054	1.000 (0.999, 1.002)	0.57025
LMR	0.782 (0.682, 0.895)	0.00038	0.890 (0.769, 1.030)	0.11742	0.903 (0.783, 1.042)	0.16184
SII	1.0001 (1.0001, 1.0002)	0.00019	1.0001 (1.0000, 1.0002)	0.030095	1.0001 (1.0000, 1.0002)	0.048459
SIRI	1.045 (1.027, 1.063)	<0.00001	1.035 (1.016, 1.055)	0.00029	1.032 (1.012, 1.053)	0.00152
Neutrophil	1.103 (1.068, 1.139)	<0.00001	1.106 (1.061, 1.152)	<0.00001	1.103 (1.058, 1.150)	<0.00001
Lymphocyte	0.759 (0.608, 0.947)	0.01465	0.857 (0.656, 1.120)	0.25798	0.878 (0.671, 1.150)	0.34385
Monocyte	1.489 (1.044, 2.124)	0.02787	1.614 (1.050, 2.481)	0.02923	1.508 (0.970, 2.344)	0.06799
PLT	0.997 (0.995, 0.999)	0.015	0.997 (0.994, 1.000)	0.02425	0.997 (0.994, 1.000)	0.03301

NLR tertile	Crude	*p* value	Model I	*p* value	Model II	*p* value
	HR (95% CI)		HR (95% CI)		HR (95% CI)	
Low	1 (ref)		1 (ref)		1 (ref)	
Middle	2.770 (1.742, 4.405)	0.00002	2.579 (1.518, 4.384)	0.00046	2.591 (1.515, 4.432)	0.00051
High	4.492 (2.867, 7.039)	<0.00001	3.549 (2.076, 6.068)	<0.00001	3.520 (2.039, 6.077)	<0.00001
*p* for trend test	2.034 (1.649, 2.508)	<0.00001	1.821 (1.413, 2.348)	<0.00001	1.815 (1.401, 2.352)	<0.00001

Adjust model I adjust for: age, sex, weight, race, and marital status; adjust model II adjust for: age, sex, weight, race, marital status, hypertension, diabetes, hyperlipidemia, and GCS score.

**Figure 4:**
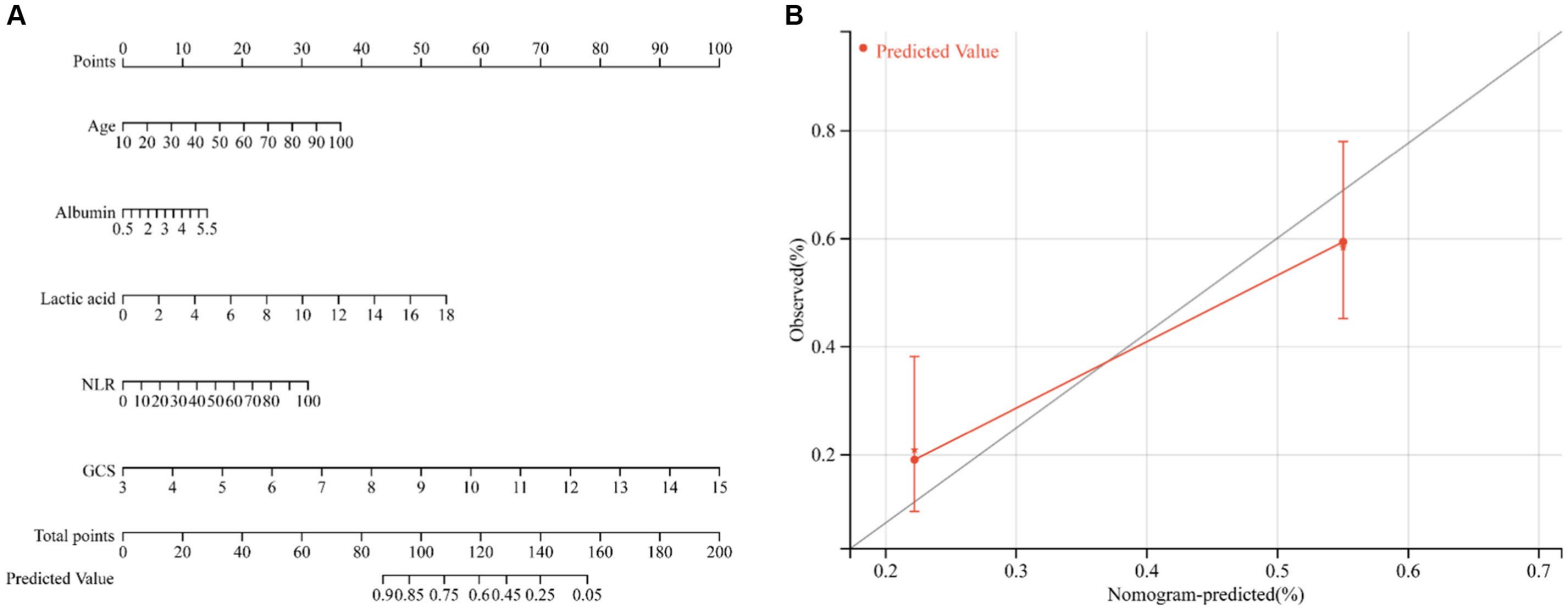
Nomogram to estimate the risk of ICU mortality in patients with ICH **(A)**. The calibration curve was used to evaluate the utility of the nomogram **(B)**.

Based on the results of multivariate logistic regression analysis, a nomogram was developed to predict the risk of ICU death in patients with ICH. Using age, albumin, lactic acid, NLR, and GCS score as variables, the calibration curve demonstrated good consistency between predicted and actual probabilities (C-index: 0.694, 95%CI: 0.643–0.745), indicating acceptable accuracy in predicting ICU mortality (Figure 4).

### Subgroup analysis

**Figure 5:**
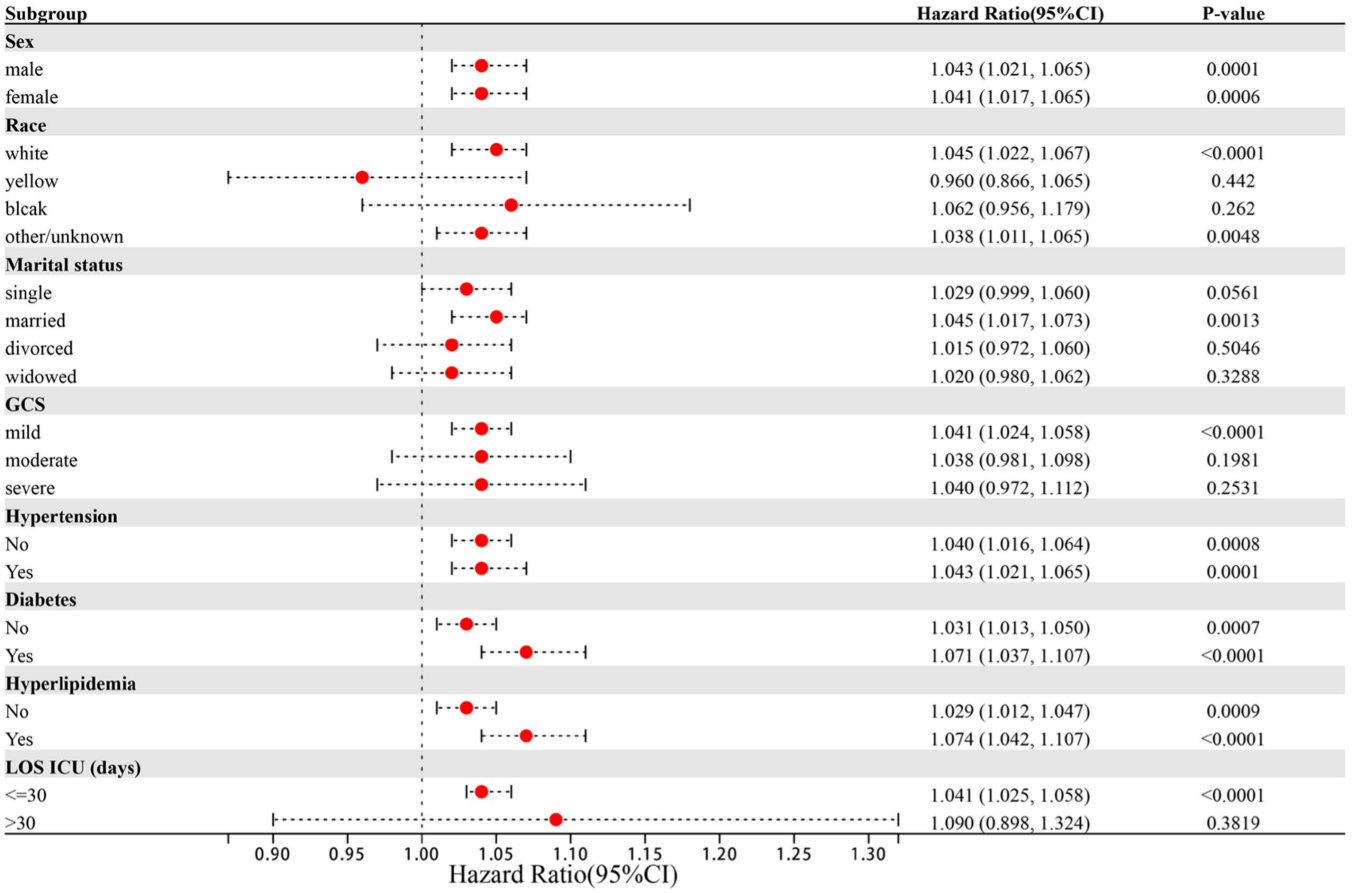
Forest plots of hazard ratios for the ICU mortality in different subgroups. HR, hazard ratio; CI, confidence interval.

The results of further stratified analyses indicate a significant association between the NLR index and a higher risk of in-hospital ICU mortality in specific subgroups of patients with ICH (Figure 5). The subgroups that showed a significant association include patients who were married (HR: 1.045, 95%CI: 1.017–1.073), of white ethnicity (HR: 1.045, 95%CI: 1.022–1.067), and those with a GCS score indicating mild coma (HR: 1.041, 95%CI: 1.024–1.058). Notably, subgroup analysis based on sex, hypertension, diabetes mellitus, and hyperlipidemia did not yield significant results, likely due to the predominantly older adult population in this study.

## Discussion

In clinical practice, ICH is a prevalent critical disease associated with a high mortality rate (20). This prospective study aims to assess the association between inflammatory indexes and clinical outcomes in critically ill patients with ICH focusing on inflammatory immune cells. For the first time, we conducted a comparative analysis of the predictive value of NLR, PLR, LMR, SII, and SIRI for ICU mortality risk in patients with ICH. The results revealed that NLR, SIRI, and LMR exhibited high predictive value for ICU mortality, with NLR demonstrating a significant association with ICU mortality in patients with ICH. These findings are anticipated to enhance early prediction and identification of ICU mortality in ICH patients.

During the process of ICH, the rupture of blood vessels and subsequent formation of hematoma initiate a range of inflammatory and immune responses. These include the activation of inflammatory cells as well as the release of inflammatory mediators (21). The hematoma triggers the activation of microglia, while immune cells such as neutrophils, monocytes, and lymphocytes from the peripheral blood are recruited through the disrupted blood–brain barrier (BBB). Consequently, they rapidly infiltrate the brain tissue, each exerting their distinct roles in the occurrence and progression of neuroinflammation (22). Previous studies have provided evidence linking leukocytosis with severe disability, unfavorable neurological outcomes, and increased mortality rates (23, 24). In terms of neutrophils and monocytes, their accumulation both in the bloodstream and at the site of injury represents a crucial characteristic of inflammation, serving as a response to either infection or the intensity of the inflammatory process (25). Lymphocytes, on the other hand, act as primary regulators of the immune system and hold a significant role in defending the host against pathogens. A decrease in lymphocyte count results in weakened immune capabilities and heightened susceptibility to infections (26, 27). Furthermore, abnormalities in platelet function directly reflect the coagulation response following ICH, thus influencing its prognosis (28, 29).

In this study, we conducted an investigation into the forecasting of five indicators to assess the risk of mortality in ICU for ICH patients. The outcomes demonstrated that the following indicators, namely, NLR, SIRI, SII, and LMR, exhibited strong predictive capabilities, with an AUC greater than 0.600. Through the process of adjusted multivariate logistic regression analysis, it was determined that NLR, SIRI, and SII independently contributed to the risk factors, but subsequent comparison revealed that NLR presented the highest predictive ability compared to the other four indicators. Therefore, it is crucial to calculate the values of NLR, SII, and SIRI upon admission for critically ill ICH patients, with particular emphasis on NLR, as a means to establish early intervention strategies and ensure a favorable prognosis for patients with severe ICH. It should be noted that SII and SIRI serve as novel inflammatory biomarkers. SIRI effectively reflects the overall homeostasis between inflammatory response and immune function, while SII integrates thrombotic activity, inflammatory response, and adaptive immune response to identify high-risk individuals who are likely to experience unfavorable outcomes or death subsequent to ICH (30–32). Nevertheless, it is worth mentioning that the predictive capacity of SII on mortality risk is affected by the time frame in which the baseline blood sample is obtained. Multiple studies have confirmed that a median SII value at approximately 29 h after ICH onset corresponds to the prognosis of 90 days after, whereas the baseline SII value in our research was obtained within 24 h, thus leading to a potential discrepancy due to a certain lag (33). NLR and SIRI exhibit substantial similarities in terms of their components, yet NLR surpasses SIRI in its ability to predict ICU mortality in cases of ICH. Additionally, this study delved into a comprehensive analysis of the risk stratification within each subgroup. Our subgroup analysis revealed that the predictive value of NLR concerning ICU mortality risk remained consistent across both male and female patients. Nevertheless, more attention should be given to factors such as marital status, race, and the Glasgow Outcome Scale. While the Glasgow Coma Scale is commonly used to assess the severity of intracerebral hemorrhage, it is important to also consider cases of mild coma. In this study, we found that racial differences also impact the risk of ICU death. This can be attributed to genetic variations between races, which may influence the structure and function of cerebral vasculature (34). Additionally, lifestyle differences, such as high-salt and high-fat diets, along with unhealthy habits like alcohol and tobacco use, can contribute to conditions such as diabetes, high cholesterol, and obesity, thereby increasing the risk of ICH death (35). Similarly, differences in social roles and the levels of stress individuals experience can also affect the risk of death from ICH. Married patients often play crucial roles in their families, such as caring for children, spouses, and parents. The physical stress from these responsibilities, the financial pressures of earning money, and the psychological stress from marital discord can negatively impact their health, leading to a higher risk of ICH and a worse prognosis (36, 37). Epidemiological studies suggest that marital instability may reduce patients’ ability to prevent and treat diseases, potentially increasing the risk of death from ICH (38, 39). Additionally, studies have confirmed that adults who remarry experience more serious physical and psychological diseases than those in stable and healthy marriages (40, 41). Therefore, maintaining a healthy and stable marriage can help patients reduce anxiety and stress, promote active treatment, and lower the risk of death from ICH.

In the study, we unexpectedly found that lactic acid and albumin were also independent risk factors for ICU mortality risk in ICH. Lactic acid is widely regarded as an indicator of tissue hypoperfusion and has been shown to be closely related to mortality in severe diseases such as sepsis, shock, and trauma (42–44). In the non-surviving patients, lactic acid levels were significantly higher than in the surviving group. Hematoma compression of the brain parenchyma leads to insufficient blood flow, which in turn causes astrocytes to take up glucose and metabolize it into lactic acid under the stimulation of large amounts of glutamate. The generated lactic acid is transferred to neurons and enters the tricarboxylic acid cycle to meet the energy demand of the brain (45, 46). Although higher serum lactic acid is beneficial for energy supplementation, the adverse pathophysiological conditions indicated by higher serum lactic acid may have a greater impact on outcomes than the relatively transient and small effects of energy supplementation. Albumin is produced by hepatocytes to maintain the physiological functions of the healthy body in various ways. Low albumin levels are also considered a valid marker of malnutrition (47). In this study, the albumin level of the non-survivors was obviously lower than that of the survivors, which further confirmed the decrease of albumin after ICH and its relationship with mortality (48, 49). Based on this, the present study demonstrates the utility of age, albumin, lactic acid levels, NLR value, and GCS score as predictive variables for assessing the risk of ICU mortality among patients with ICH. This model holds particular promise in facilitating timely symptomatic interventions and serving as a warning system for critically ill patients with ICH to prevent disease progression and avoid the deterioration of the disease.

This study has some limitations that need to be acknowledged. Firstly, we lacked information on whether patients with ICH underwent surgical treatment or not. Secondly, patients’ personal histories, including smoking and alcohol consumption, were not available, and their nutritional status or the severity of comorbid conditions were not captured in the database. Additionally, we only recorded inflammatory markers for the first 24 h after admission. The inflammatory response may evolve over several days following the onset of ICH. Therefore, further investigations are warranted to explore the changes in inflammatory factors at different time points after onset and their predictive value.

## Conclusion

In conclusion, our study reveals that NLR, SIRI, and LMR serve as effective predictors of ICU mortality risk in patients with ICH. Notably, NLR exhibits superior predictive performance. Simultaneously, constructing a nomogram with NLR, lactic acid, and albumin as variables proves beneficial for clinical decision-making and disease management, mitigating the risk of ICH deterioration.

## Data availability statement

Publicly available datasets were analyzed in this study. This data can be found at: doi: 10.13026/6mm1-ek67.

## Ethics statement

Ethical review and approval was not required for the study on human participants in accordance with the local legislation and institutional requirements. Written informed consent from the [patients/participants OR patients/participants legal guardian/next of kin] was not required to participate in this study in accordance with the national legislation and the institutional requirements.

## Author contributions

GZ: Writing – original draft, Writing – review & editing, Data curation, Methodology, Resources, Software. YG: Data curation, Writing – review & editing. ZW: Methodology, Software, Writing – review & editing. YC: Formal analysis, Validation, Writing – review & editing. XX: Writing – review & editing, Formal analysis, Validation.
